# Infection with Human T Lymphotropic Virus 1 is associated with bronchiectasis among Indigenous Australians

**DOI:** 10.1186/1742-4690-8-S1-A38

**Published:** 2011-06-06

**Authors:** Lloyd Einsiedel, Liselle Fernandes, Tim Spelman, Eduardo Gotuzzo

**Affiliations:** 1 Department of Medicine, Alice Springs Hospital, Northern Territory, 0870, Australia; 2 Northern Territory Rural Clinical School, Alice Springs, Northern Territory, 0870, Australia; 3 Department of Medicine, Cayetano Heredia University, Lima, Peru

## Background

Infection with the Human T-Lymphotropic Virus 1 (HTLV-1) is associated with bronchiectasis in Indigenous Australians [1]. The present study defines the clinical presentation and outcomes of bronchiectasis according to HTLV-1 serostatus in this population.

## Materials and methods

Retrospective cohort study at Alice Springs Hospital, central Australia. Medical records were reviewed for all Indigenous adults admitted between 2000-2006 with radiologically confirmed bronchiectasis and known HTLV-1 serostatus.

## Results

One-hundred and twenty patients were admitted during the study period, HTLV-1 serology was performed for 92 (75.8%) patients. Western blots confirmed HTLV-1 infection in 52 (58.4%) cases and were indeterminate for 3 patients. HTLV-1 seropositive patients more often had bilateral bronchiectasis (HTLV-1+, 37/51; HTLV-1-, 18/36; p=0.032) and ground glass opacities (HTLV-1+, 10/51; HTLV-1-, 1/36; p=0.028) on HRCT chest, but were less likely to have a pathogen isolated during an infective exacerbation (HTLV-1+, 16%; HTLV-1-, 20%; p=0.042). HTLV-1 seropositive patients were more likely to have cor pulmonale (HTLV-1+, 10/52; HTLV-1-, 1/37; p=0.023) and to suffer bronchiectasis-related deaths (OR 5.78; 95% CI, 1.17, 26.75; p=0.028). The mortality rate for the entire cohort during the 7 year period was 34.2%. Median age of death for both groups combined was 42.5 years. Only HTLV-1 seropositive patients were admitted for treatment of infected skin lesions and this was the major predictor of subsequent death from any cause on multivariable analysis (OR, 6.77, 95% CI, 1.46, 31.34; p=0.014).

## Conclusion

In an Indigenous Australian cohort HTLV-1 infection is associated with bronchiectasis and an increased risk of pulmonary hypertension and death.
